# LUCID: Intelligent Informative Frame Selection in Otoscopy for Enhanced Diagnostic Utility

**DOI:** 10.3390/diagnostics16182981

**Published:** 2026-09-15

**Authors:** Hao Lu, Muhammet F. Demir, Gabriella I. Puchall, Zian Shang, Tucker Corwen, Shalaka Chavan, Carl D. Langefeld, Amy Zinnia, Delal Seker, Muhammad Khalid Khan Niazi, Aaron C. Moberly, Metin N. Gurcan

**Affiliations:** 1 Center for Artificial Intelligence Research, Wake Forest University School of Medicine, Winston-Salem, NC 27101, USA; muhammet.demir@advocatehealth.org (M.F.D.); gabriella.puchall@wfusm.edu (G.I.P.); shalaka.chavan@advocatehealth.org (S.C.); metin.gurcan@wfusm.edu (M.N.G.); 2 Department of Biostatistics and Data Science, Wake Forest University School of Medicine, Winston-Salem, NC 27101, USA; carl.langefeld@wfusm.edu (C.D.L.); amy.heney@advocatehealth.org (A.Z.); 3 Department of Electrical and Electronics Engineering, Dicle University, Diyarbakir 21280, Turkey; delal.seker@dicle.edu.tr; 4 Department of Pathology, The Ohio State University, Columbus, OH 43210, USA; khalid.niazi@osumc.edu; 5 Department of Otolaryngology–Head and Neck Surgery, Vanderbilt University Medical Center, Nashville, TN 37232, USA; aaron.c.moberly@vumc.org

**Keywords:** informative frame selection, otoscopy, middle ear diagnosis, deep learning, eardrum segmentation, image quality assessment

## Abstract

**Background/Objectives**: Accurate diagnosis of middle ear disease remains challenging because otoscopic interpretation is subjective, while many artificial intelligence models depend on manually selected still frames from video examinations. We aimed to develop and internally evaluate LUCID, a systematic framework for automatically identifying the most informative frames in otoscopy videos. **Methods**: We analyzed 713 otoscopy videos from 491 patients. LUCID combines a ResNet-50 classifier for coarse eardrum-visibility assessment, BC-AdvCAM for weakly supervised eardrum localization and coverage estimation, and an otoscope-specific blur/focus assessment into a within-video frame-ranking score. Automated selections were compared with expert-selected frames through blinded human review and downstream classification. To separate ranking quality from the effects of bag size and multi-frame aggregation, a matched 12-frame control using uniform temporal sampling was evaluated with the same ResNet-50 and attention-based multiple instance learning (ABMIL) pipeline. **Results**: Among 293 video pairs with complete ratings, algorithm-selected frames were rated equal to or better than expert-selected frames in 74.7% of evaluations on average. However, when an evaluator expressed a non-Equal preference, the expert frame was favored in 71.7% and 70.7% of cases by the two evaluators, respectively; among concordant non-Equal ratings, 80.0% favored the expert frame. BC-AdvCAM achieved an intersection over union of 0.6717 and a Dice similarity coefficient of 0.7924 on a held-out manually segmented test subset. For downstream diagnosis, patient-level mean accuracy for the matched single-frame comparison was 68.79% for LUCID Top-1 versus 72.17% for the expert-selected frame (difference, −3.38 percentage points; 95% exact sign-flip-inversion CI, −6.79 to 0.00; two-sided exact patient sign-flip *p* = 0.0540). In the matched 12-frame comparison, LUCID Top-12 achieved 78.13% versus 59.41% for Uniform-12 using the identical ABMIL pipeline (difference, +18.71 percentage points; 95% exact sign-flip-inversion CI, +15.79 to +21.63; two-sided exact patient sign-flip *p* = 2.33 × 10^−10^). **Conclusions**: In the matched single-frame comparison, the expert-selected frame had the higher observed patient-level mean accuracy, whereas LUCID provided an automated ranking of the full video that substantially improved matched 12-frame ABMIL performance relative to uniform temporal sampling. These results support LUCID as an automated frame-ranking and multi-frame evidence-selection tool, while external validation remains necessary before clinical deployment.

## 1. Introduction

Middle ear diseases are among the most common conditions encountered in clinical practice. Yet, accurate diagnosis remains challenging, as physicians often rely on subjective visual assessment through otoscopy. Even experienced primary care, emergency medicine, and otolaryngology (ENT) physicians struggle to achieve high diagnostic accuracy, with reported accuracy rates ranging from a modest 50% to 80%, even when utilizing pneumatic otoscopy [1,2]. This issue is particularly pronounced among clinicians with less otoscopy training, who account for the vast majority of clinical visits for ear complaints. For context, there are over 400,000 primary care clinicians in the U.S., compared to only 12,609 ENT physicians [3,4].

To address the challenge of low diagnostic accuracy, many recent efforts have focused on developing machine learning (including deep learning) models to assist clinicians [5,6,7,8,9,10,11,12,13]. While these studies demonstrate promising image-level diagnostic performance, many systems still depend on selected still images rather than explicitly ranking the diagnostic utility of frames within an otoscopy video. Clinical examinations are inherently dynamic, and a recorded examination may contain incomplete tympanic-membrane views, motion blur, glare, obstruction, and redundant content. Automated selection of informative frames can therefore support more efficient review and provide standardized inputs for downstream analysis [14].

However, identifying MIFs in otoscopy videos poses unique challenges compared with generic video summarization or video quality assessment tasks. Otoscopy image interpretation requires specialized medical expertise, making labeled data extremely scarce. Two experienced ENT specialists jointly reviewed each of 303 evaluation videos and selected one reference MIF by consensus. This set was sufficient for evaluation but not for large-scale supervised training. Consequently, our framework leverages weakly supervised learning and knowledge-based decision strategies to reduce dependence on large-scale expert frame annotations.

In this work, we introduce LUCID (Leveraging Useful Content in Informative Diagnostics), a systematic framework for automatic MIF selection in otoscopy videos. LUCID evaluates each frame based on three complementary visual dimensions: (1) eardrum visibility, (2) eardrum coverage, and (3) image clarity. Specifically, we train a ResNet-50 model to assess eardrum visibility, employ a weakly supervised BC-AdvCAM method for estimating eardrum coverage, and design an otoscope-specific blur and focus detection module to quantify image clarity. The resulting features are integrated into a unified informative score used to rank frames within each video.

Evaluations involving expert reviewers and multiple deep learning architectures showed that expert selection remained competitive for the single-frame task. We therefore distinguish single-frame selection from multi-frame evidence construction: the former is evaluated by the matched Top-1-versus-Human comparison, whereas the latter is evaluated using a matched 12-frame Uniform-12 control with the identical ABMIL architecture. This design allows the effect of LUCID ranking to be separated from the effect of simply providing more frames to a multi-frame model.

LUCID contributes a domain-specific integration of BC-AdvCAM, otoscope-specific blur-focus analysis, and a unified informativeness metric designed to prioritize diagnostically useful frames rather than generic image quality alone. The contribution of this study is the design and evaluation of this integrated otoscopy-specific pipeline. We explicitly position LUCID relative to earlier frame-filtering, frame-selection, video-representation, and endoscopic image-quality approaches rather than making a priority claim. Our key contributions are:

- Development of a labeled dataset of 38,825 frames categorized by eardrum visibility for ResNet-50 training.
- The proposal of BC-AdvCAM, a weakly supervised model enabling eardrum segmentation with only image-level labels.
- Design of an otoscope-specific blur and focus detection method integrated into a unified informative scoring framework.
- Empirical evaluation against expert-selected frames and downstream diagnostic models, including a matched single-frame comparison and a matched 12-frame uniform-sampling control for multi-frame aggregation.
- Introduction of an automated pre-annotation workflow that may assist expert labeling and future otoscopic AI dataset development.

The remainder of this paper is organized as follows: Section 2 reviews still-image otoscopy diagnosis, otoscopy video analysis and frame selection, informative-frame selection in related medical endoscopy, and video quality assessment. Section 3 describes the methodology for eardrum view, coverage, blur detection, informative scoring, and the experimental setup. Section 4 presents quantitative results comparing expert-selected frames with those selected by the algorithm. Section 5 discusses the findings in the context of prior frame-selection approaches, limitations, and implications. Section 6 concludes the paper and outlines future directions.

## 2. Related Work

### 2.1. Still Image-Based Diagnostic Assistance

Many studies have explored high-quality static otoscopy images for computer-assisted diagnosis [5,6,7,8,9,10,11,13]. These approaches primarily use convolutional or transformer-based classifiers to identify middle-ear abnormalities from manually captured or selected images. More recently, Ear-Keeper demonstrated large-scale, multi-center otoscopic image classification with external validation and cross-platform real-time deployment [12]. These results show substantial progress in image-level diagnosis, but image classifiers do not by themselves determine which frames within a heterogeneous examination video contain the most useful diagnostic evidence. This distinction motivates methods that explicitly filter, select, rank, or aggregate video frames.

### 2.2. Otoscope Video-Based Diagnosis and Frame Selection

Several studies have addressed otoscopy video analysis using different strategies. SelectStitch uses tympanic-membrane segmentation to retain frames containing a sufficient eardrum area and then stitches the selected frames into a composite image [15]. OtoXNet represents a video using multiple composite images and demonstrated improved disease-classification accuracy relative to a single composite or a manually selected keyframe [16]. VideoMAE instead learns directly from sampled video clips, avoiding explicit manual frame selection but without producing an interpretable MIF ranking [17]. Wang et al. used a supervised eardrum detector to retain valid frames and then aggregated frame-level anomaly scores for pediatric normal-versus-abnormal video screening [18]. A clinical reader study further compared computer-generated SelectStitch composites, manually selected still frames, and full videos, illustrating the trade-off between compact visual summaries and review time [14]. Most recently, OtoSTVT introduced diagnostic-driven frame selection using a spatio-temporal transformer trained with ordinal frame-relevance labels and contrasted its end-to-end learned strategy with LUCID’s knowledge-guided design [19]. These studies establish that automatic frame filtering and video representation in otoscopy predate the present work and provide important direct comparators for LUCID.

### 2.3. Informative Frame Selection in Related Medical Endoscopy

Informative-frame selection has also been studied in other endoscopic domains. In flexible laryngoscopy, Yao et al. trained a ResNet-18 classifier from reviewer labels based on vocal-fold visibility, illumination, focus, and camera distance, achieving an informative-frame F1-score of 92.3% [20]. Baldini et al. subsequently demonstrated real-time informative-frame classification with independent external validation across white-light and narrow-band laryngoscopy images [21]. A 2026 study by Kim et al. extended this direction to three-level laryngoscopic image-quality assessment and externally validated high-quality-image selection on the Laryngoscope8 dataset [22]. In wireless capsule endoscopy, Li et al. addressed a related but distinct objective by using a multi-scale triplet network to remove redundant frames in very long videos while preserving clinically useful content [23]. Together, these studies show that visibility, focus, image quality, redundancy, and diagnostic relevance are complementary concepts rather than interchangeable definitions of informativeness.

### 2.4. Video Quality Assessment

The task of selecting informative frames is also related to natural video quality assessment (VQA). VQA research broadly distinguishes subjective quality assessment, which relies on human judgment [24,25,26,27], from objective assessment [28,29,30,31,32,33]. Objective methods may be Full-Reference, Reduced-Reference, or No-Reference (NR), depending on whether a reference image or video is available. Because otoscopy examinations do not provide an ideal reference frame, the image-quality component of LUCID is most closely related to the NR setting. However, perceptual quality alone is insufficient for MIF selection because a visually sharp frame may still omit the tympanic membrane or provide inadequate diagnostic coverage.

NR quality assessment includes both knowledge-driven approaches based on handcrafted perceptual features [34,35] and deep-learning approaches that learn quality-related representations from labeled data [36,37,38]. LUCID does not attempt to replace general VQA; instead, it uses image clarity as one component within a medical-task-specific ranking that also accounts for tympanic-membrane visibility and coverage.

Accordingly, our specific challenge extends beyond generic image quality: a selected frame must show the relevant anatomy at sufficient scale and clarity for otoscopic interpretation. LUCID therefore uses a hybrid strategy that combines learned eardrum-view estimation, weakly supervised localization, and explicit blur/focus analysis. This design reduces dependence on dense expert frame-relevance labels while retaining interpretable component scores, but it does not model temporal relationships between frames as explicitly as end-to-end spatio-temporal methods such as OtoSTVT [19].

## 3. Materials and Methods

**Figure 1:**
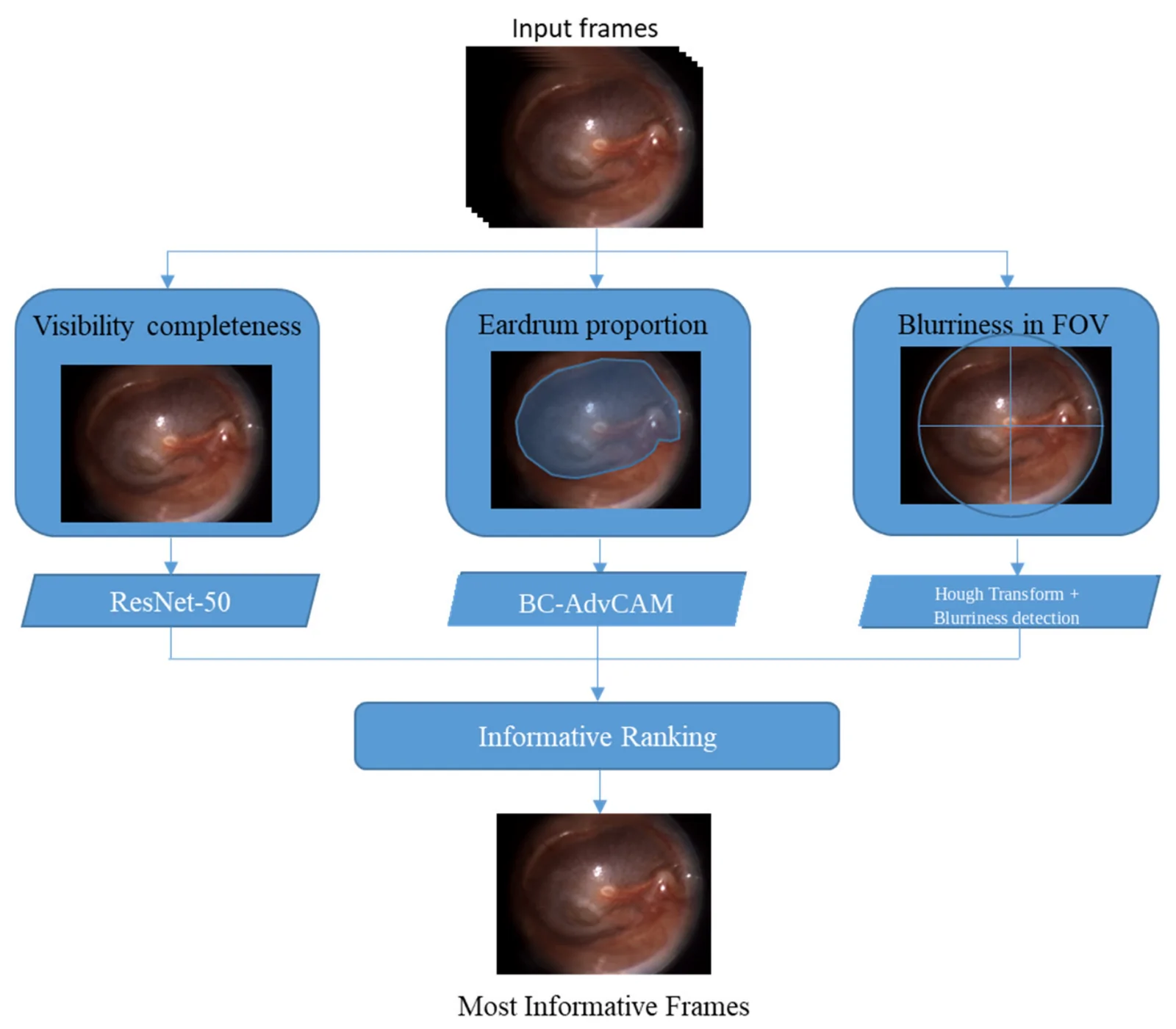
Most Informative Frame (MIF) selection pipeline.

This section details the proposed methodology for identifying diagnostically important frames from otoscope video sequences. The method systematically addresses three primary challenges that commonly render frames diagnostically unimportant: partial visibility of the eardrum, low eardrum coverage, and image blur. The overall workflow of this process is illustrated in Figure 1**.** Specifically, each video frame is first analyzed by a ResNet-50 classifier to estimate the visibility completeness of the tympanic membrane—whether the entire eardrum is clearly visible in the frame. Next, a weakly supervised segmentation is performed using our proposed BC-AdvCAM method to estimate the proportion of the eardrum region within the image. In parallel, a Hough Transform is applied to detect the circular field-of-view (FOV) and determine the focal point. A blurriness score is then computed based on the distance between the focus point and the FOV center, as well as the overall blur level within the FOV. Finally, the visibility completeness, eardrum coverage ratio, and blurriness score are jointly integrated to rank all frames by their informativeness, and the top-k ranked frames are selected as the most informative frames for downstream disease classification.

### 3.1. Dataset Acquisition and Annotation

The dataset utilized in this study comprises 713 high-definition otoscope videos from 491 patients, with each video representing a distinct ear examination. Because one or both ears could be examined, a single patient could contribute one or two videos. These videos were collected from adult and pediatric ear, nose, and throat (ENT) clinics and operating rooms. Ethical approval for the study protocol was obtained from the Wake Forest University Health Sciences Institutional Review Board (IRB0004833), with data collection conducted after written informed consent was obtained from patients and/or their guardians.

All video recordings were captured using the JEDMED Horus+ HD Video Otoscope (JEDMED, St. Louis, MO, USA), a high-definition digital otoscope known for its capability to acquire high-quality otoscopic imagery. Videos were stored in MPEG-4 format with a resolution of 1440 × 1080 pixels. Ground truth diagnoses for each case were rigorously established by a board-certified ENT specialist. This diagnostic process relied on microscopy in the clinic or operating room as the gold standard, further complemented by assessments of hearing status and eardrum mobility derived from audiometry and tympanometry.

All dataset partitions were constructed at the patient level so that all videos from the same patient remained within a single partition. The 713 videos were obtained from 491 patients (400 adults and 91 pediatric patients) and were divided into a 410-video LUCID development cohort from 296 patients (241 adults and 55 pediatric patients) and a separate 303-video LUCID evaluation cohort from 195 patients (159 adults and 36 pediatric patients), with no patient overlap between the two cohorts. The development cohort was further divided into 296 training videos from 207 patients (169/38 adult/pediatric), 63 validation videos from 44 patients (36/8), and 51 test videos from 45 patients (36/9). For the downstream classification experiments, the evaluation cohort was divided into 227 training videos from 142 patients (116/26), 30 validation videos from 19 patients (15/4), and 46 test videos from 34 patients (28/6). Diagnostic category distributions are summarized in Table 1. Partitioning was also enforced at the video level: all frames from a given video were retained within the same partition, so adjacent or near-duplicate frames from one video could not appear across training, validation, and test sets.

For evaluation purposes, two ENT experts jointly reviewed every video in the separate 303-video evaluation cohort and selected a single reference MIF by consensus. Because the final reference frame was determined by consensus rather than by two independent MIF annotations, inter-rater reliability was not calculated for the ENT reference selection. The 410-video development cohort was used to train and internally evaluate the frame-level components of LUCID. Three medical students first completed a one-week otoscopy training program. The annotation workload was then divided among the three students, and each student separately annotated an assigned subset of frames as “no eardrum,” “partial eardrum,” or “full eardrum,” for a total of 38,825 annotated frames. Because the frames were not redundantly annotated by all three students, inter-rater reliability could not be estimated for these frame-level labels. These annotations were used to train the eardrum-view classifier and weakly supervised eardrum segmentation model. For the three-class eardrum-view annotation, frames with visually ambiguous partial-versus-full visibility were excluded rather than forced into a category; the retained labels therefore represent a deliberately coarse visibility-quality task with unambiguous class membership.

### 3.2. Eardrum View Score: Addressing Partial Eardrum Visibility

As we mentioned, frames often present a partial or absent view of the eardrum, which contains less diagnostic information. To quantify the diagnostic importance related to eardrum visibility, we developed an “Eardrum View Score” based on a deep learning classification. We labeled 38,825 frames from 410 videos into three categories:

Full Eardrum: The eardrum is completely visible within the frame.

Partial Eardrum: Only a portion of the eardrum is visible.

No Eardrum: The eardrum is entirely absent from the frame.

We use these data to train a ResNet-50 model.

Borderline frames in which a substantial portion of the tympanic membrane was visible but the distinction between “partial” and “full” visibility remained uncertain were excluded during annotation. This exclusion was intentional because the Eardrum View Score was designed as a coarse quality-control component rather than a fine-grained clinical grading task.

Based on the class posterior probabilities produced by the ResNet-50 model, we compute a continuous measure of tympanic-membrane visibility and completeness. The three classes are assigned utilities of 1.0 (full eardrum), 0.5 (partial eardrum), and 0.0 (no eardrum).

Full Eardrum: Score of 1.0

Partial Eardrum: Score of 0.5

No Eardrum: Score of 0.0

The Eardrum View Score, *S_edv_*, for a frame is the expected utility under the model-predicted class probabilities:

*S_edv_* = *P_full_* × 1 + *P_partial_* × 0.5 + *P_non_* × 0

where *P_full_*, *P_partial_*, and *P_non_* denote the predicted probabilities of the full-eardrum, partial-eardrum, and no-eardrum classes, respectively. The probabilities sum to one, and *S_edv_* ranges from 0 to 1; larger values indicate a more complete tympanic-membrane view.

### 3.3. Eardrum Coverage Score: Addressing Low Eardrum Coverage

Low coverage occurs when the eardrum occupies a small fraction of the total frame area, often due to a wide field of view, making detailed examination difficult. To address this, we need to segment the eardrum pixels. However, obtaining pixel-level eardrum labels requires significant expert effort, rendering ground truth annotation very expensive. Hence, we employ a weakly supervised semantic segmentation method that uses only image-level labels (indicating the presence or absence of an eardrum). We also used the data labeled as “no eardrum,” “partial eardrum,” and “full eardrum” from Section 3.2. By grouping “partial eardrum” and “full eardrum” into a single “presence eardrum” category, we formulated a pixel-level binary classification problem.

#### 3.3.1. Modified AdvCAM (BC-AdvCAM) for Weakly Supervised Semantic Segmentation

To perform Weakly Supervised Semantic Segmentation (WSSS), we adapted the AdvCAM [39] method. AdvCAM is designed to enhance Class Activation Maps (CAMs) for weakly supervised semantic segmentation. Traditional CAMs tend to focus only on the most discriminative regions of an object, which are sufficient for classification but fail to capture the full extent of the target object. AdvCAM addresses this limitation by employing adversarial climbing to expand object regions and regularization to suppress irrelevant activations, resulting in dense and accurate localization maps.

Adversarial climbing is the core of AdvCAM, which manipulates an input image iteratively to maximize the classification score for a target class. Unlike adversarial attacks that aim to deceive the model, adversarial climbing modifies the image to amplify the contribution of non-discriminative regions relevant to the target class.

The process begins with an input image *x*_0_ and iteratively updates it using Equation (1):

(1) $$x_{t}\;=\;x_{t-1}+\varepsilon \nabla_{x_{t-1}}L,$$

where:

(2) *L* = *y_c_* − *L_class_*_−_*_suppress_* − *L_restrict_*,

(3) $$L_{\mathrm{class}-\mathrm{suppress}}=-\sum_{k\in C\backslash c}y_{k},$$

(4) $$L_{\mathrm{restrict}}=\lambda {\left\|M⊙\mathrm{CAM}\left(x_{t}\right)-\mathrm{CAM}\left(x_{0}\right)\right\|}_{1},$$

(5) *M* = 1(*CAM*(*x_t_*_−1_) > *τ*),

In Equations (1)–(5), *x_t_* denotes the perturbed input at adversarial-climbing iteration *t*, with *x*_0_ denoting the original input. The scalar epsilon is the step size, and the gradient is taken with respect to the current input. The term *y_c_* is the classification score (logit) for target class *c*, while *C* denotes the set of all classes and *y_k_* denotes the logit for class *k*. *L* is the overall adversarial-climbing objective; *L_class−suppress_* suppresses competing class responses and *L_restrict_* limits changes in regions that are already highly activated. *CAM*(*x_t_*) denotes the class activation map at iteration *t*. *M* is the binary restriction mask, tau is the activation threshold used to construct *M*, 1(.) is the indicator function, the symbol ⊙ denotes element-wise multiplication, and lambda is the regularization weight applied to *L_restrict_*.

This iterative process ensures that non-discriminative regions—those not initially involved in the classification—are gradually included in the model’s attention, thereby expanding the CAM to cover a larger portion of the target object.

The conventional AdvCAM formulation is typically used for multi-class problems; however, for our binary task, we modified its calculation to explicitly incorporate the background class, leading to more precise segmentation boundaries. The proposed binary class AdvCAM, BC-AdvCAM, formulation is as follows:

(6) *x_BC_*_−_*_ADvCAM_* = *AdvCAM*_1_(*x*) − *λ* × *AdvCAM*_0_(*x*),

where *x_BC−AdvCAM_* denotes the refined activation map used by BC-AdvCAM, *AdvCAM*_1_(*x*) is the AdvCAM activation map for the eardrum (foreground) class, *AdvCAM*_0_(*x*) is the corresponding background-class activation map, and lambda is the weighting factor controlling the contribution of background suppression in Equation (6). The refined map is then used to obtain the tympanic-membrane region for coverage estimation.

#### 3.3.2. Coverage Score Calculation

After obtaining the binary tympanic-membrane mask *M_TM_*, the Eardrum Coverage Score, *S_edc_*, is computed as the fraction of frame pixels belonging to the predicted tympanic membrane:

(7) $$S_{\mathrm{edc}}=\frac{\mathrm{eardrum}\;\mathrm{pixels}}{\mathrm{all}\;\mathrm{pixels}},$$

Here, *N_TM_* is the number of pixels belonging to the predicted tympanic-membrane region and *N_F_* is the total number of pixels in the frame, so *S_edc_* = *N_TM_*/*N_F_*. Larger *S_edc_* values indicate that the visible tympanic membrane occupies a larger fraction of the image.

### 3.4. Blur and Focus Score: Addressing Image Clarity

Blur detection in otoscope images is challenging, as traditional techniques (e.g., Fast Fourier Transform or Wavelet Transform) perform poorly. This reduced effectiveness stems from the inherent characteristics of otoscope images—specifically, their low variance and the concentration of image energy in low-frequency components. Our approach leverages edge energy distribution and a specific focus assessment to quantify image clarity.

#### 3.4.1. Edge Energy Analysis for Blur Detection

**Figure 2:**
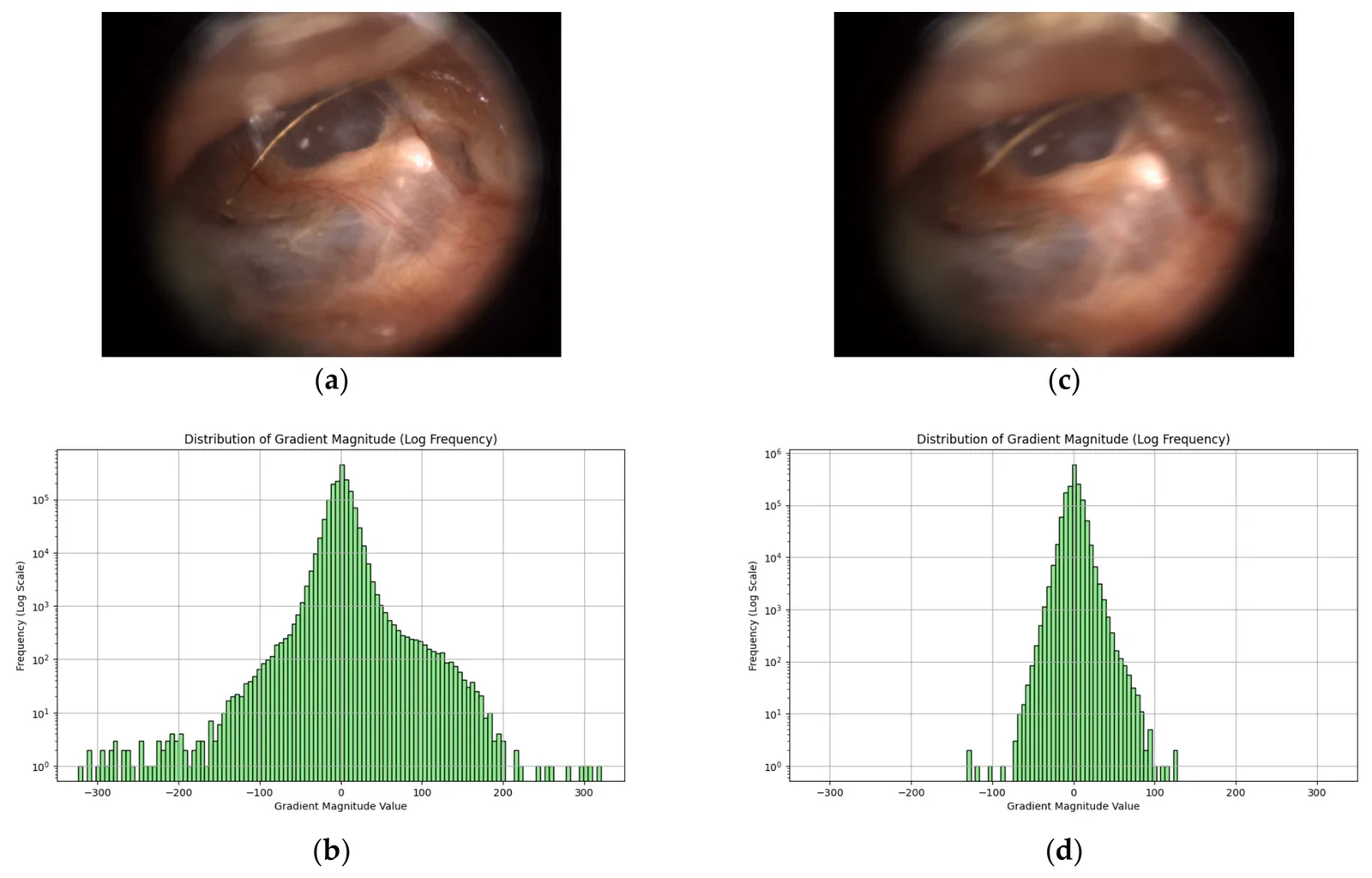
An example of the edge energy distribution for a sharp image (**a**,**b**) and a blurred image (**c**,**d**). The sign represents the edge direction.

We observed that clear otoscope images exhibit a “long tail” in their edge energy distribution, whereas blurry images show edge energy concentrated near zero, as illustrated in Figure 2. Specifically, Figure 2a,b illustrate an example of a clear (sharp) frame and its corresponding edge-energy histogram. In this example, high-magnitude edges are broadly distributed, forming a long-tailed curve that reflects abundant structural and textural details. In contrast, Figure 2c,d show the same analysis for a blurred image, where most edge energies cluster close to zero, indicating a loss of fine structural information and weak edge responses. The sign of the edge energy represents the edge direction, allowing visualization of both magnitude and orientation changes caused by image blur.

To leverage this distinction, we fit a Gaussian Mixture Model (GMM) with two Gaussian distributions to the computed edge energy distribution of a frame. A frame is deemed clear if one of the Gaussian distributions identified by the GMM possesses a large variance, indicating a broad spread of edge energies.

The initial blur score, denoted as *S_bl_*, is computed as:

(8) $$S_{\mathrm{bl}}=\frac{\sigma_{\max}}{C_{p}+\epsilon },$$

where:

*σ_max_* is the standard deviation of the Gaussian component with the larger variance in the two-component Gaussian mixture fitted to the edge-energy distribution. A larger *σ_max_* indicates a broader spread of edge responses.

*C_p_* denotes the image-contrast term used in the blur score.

epsilon is a small positive constant added to the denominator to prevent division by zero.

A larger *S_bl_* indicates a broader edge-energy distribution relative to image contrast and therefore a clearer image under this measure.

#### 3.4.2. Otoscope Field of View and Focus Point Detection

**Figure 3:**
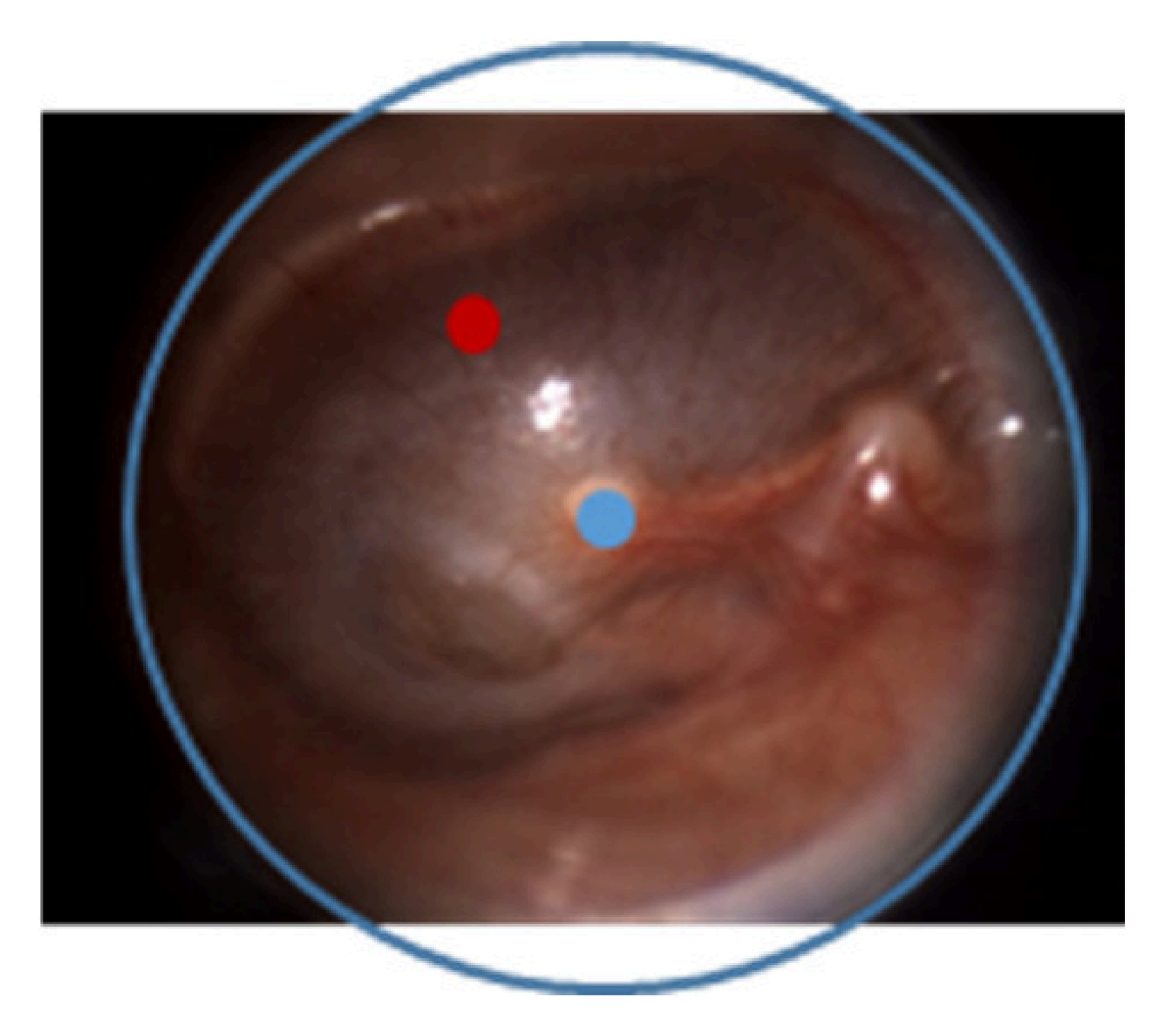
An example of FOV and focus point in one otoscope frame. The blue line shows the FOV, the blue dot is the center of the FOV, and the red dot is the focus point.

During otoscopy, the camera may not always focus precisely on the eardrum. To ensure that diagnostic attention is centered on the tympanic membrane, we first detect the otoscope’s circular field of view (FOV) and then determine how well the focus aligns with its center (Figure 3).

##### FOV Detection

We use the Hough Circle Transform to locate circular regions in the image that could represent the otoscope’s FOV. For each candidate circle, we compare the average brightness inside the circle with that of a thin surrounding ring. The circle showing the highest brightness contrast between its interior and exterior is selected as the most probable FOV, since the edge of the otoscope usually produces a sharp lightness transition.

##### Focus Point Detection

To find where the image is most sharply focused, we first smooth the image with a Gaussian filter to suppress noise and then apply the Laplacian operator, which highlights regions of rapid intensity change. The location with the strongest focus response is treated as the focus point. *S_F_* denotes the focus-alignment score derived from the proximity of this focus point to the center of the detected FOV; a higher *S_F_* indicates that the sharpest region is closer to the FOV center.

#### 3.4.3. Final Blur Score

The final Blur and Focus Score, *S_blf_*, combines the sharpness term and spatial focus term multiplicatively:

(9) *S_blf_* = *S_bl_* × *S_F_*,

In Equation (9), *S_blf_* is the final Blur and Focus Score, *S_bl_* is the edge-energy-based blur score, and *S_F_* is the focus-alignment score. A higher *S_blf_* therefore indicates a clearer frame whose sharpest region is also well aligned with the otoscope FOV.

### 3.5. Informative Score Calculation

The final Informative Score, *S_I_*, integrates the Eardrum View Score, Eardrum Coverage Score, and Blur and Focus Score using a weighted sum of their within-video ranks:

(10) *S_I_* = 0.4 × *Rank*(*S_edv_*) + 0.2 × *Rank*(*S_edc_*) + 0.4 × *Rank*(*S_blf_*),

Here, *Rank*(.) denotes the within-video rank of the corresponding component score, with better component values assigned better (smaller) ranks. The weighting factors were optimized exclusively on the LUCID development validation set comprising 63 videos from 44 patients. Specifically, a grid search was performed over w_view_, w_coverage_, w_blur−focus_ ∈{0, 0.2, 0.4, 0.6, 0.8, 1.0}, subject to w_view_ + w_coverage_ + w_blur−focus_ = 1, yielding 21 valid weight combinations. For each combination, frames were ranked using the resulting informative score, and Top-1 validation classification accuracy was used as the optimization criterion. The best-performing combination was w_view_ = 0.4, w_coverage_ = 0.2, and w_blur−focus_ = 0.4. These weights were fixed before downstream evaluation. The separate downstream classification validation set (30 videos from 19 patients) and held-out test set (46 videos from 34 patients) were not used for weight selection. In Equation (10), w_view_, w_coverage_, and w_blur−focus_ weight the contributions of the Eardrum View Score, Eardrum Coverage Score, and Blur and Focus Score, respectively.

Because better component values receive smaller rank values, a smaller *S_I_* indicates a more informative frame. The lowest *S_I_* frame is selected as Top-1, and the k frames with the lowest *S_I_* values define the Top-k set.

### 3.6. Experimental Setup

The ResNet-50 used for the Eardrum View Score was trained with 38,825 retained, unambiguous annotated frames from the 410-video development cohort. Patient- and video-level partitioning was used: 296 videos from 207 patients were assigned to training, 63 videos from 44 patients to validation, and 51 videos from 45 patients to testing. All frames from a given video remained within one split, and all videos from a given patient also remained within one split. The held-out test subset contained 13,527 frames: 1691 no-eardrum, 3317 partial-eardrum, and 8519 full-eardrum frames. Images were resized to 224 × 224 pixels, converted to tensors, and normalized using ImageNet means and standard deviations. Data loaders used a batch size of 32. A pretrained ResNet-50 was adapted to the three classes and trained for 25 epochs using Adam with a learning rate of 0.001 and cross-entropy loss. Model selection was based on the highest validation accuracy. The model used for Modified AdvCAM was trained with the same split and training protocol, except that its final layer was adapted to two classes (“no eardrum” and “with eardrum”). Foreground and background AdvCAM were each iterated 30 times.

For pixel-level segmentation evaluation, a held-out manual reference subset comprising 764 frames from 36 videos contributed by 31 patients was drawn exclusively from the test partition of the 410-video development cohort. None of these patients, videos, or frames appeared in the training or validation partitions. The selected frames included challenging conditions such as blur and glare. Manual masks were created using X-AnyLabeling. Two otolaryngologists (ACM and CE) first delineated the tympanic membrane independently on each selected frame. Their masks were then compared, and any discrepancies were discussed and jointly edited until a consensus mask was reached. The operational boundary followed the visible anatomical margin of the tympanic membrane. Only directly visible tympanic membrane was included; the boundary was not extrapolated through portions obscured by the ear canal, cerumen, or instruments. Regions substantially obscured by glare or severe blur were excluded. For partial views, only the visible and diagnostically interpretable portion of the tympanic membrane was delineated. Internal landmarks within the tympanic-membrane outline, including the malleus and umbo, were retained within the mask. These 764 frame-level observations from 31 patient clusters formed the evaluation set used for the segmentation metrics and GEE analyses.

For the downstream multi-frame analysis, we added a matched-k control to separate the effect of LUCID ranking from bag size and ABMIL aggregation. For each video, Uniform-12 selected 12 frames uniformly across the temporal extent of the video without using LUCID scores. Uniform-12 and LUCID Top-12 were evaluated with the same ResNet-50 feature extractor, ABMIL architecture, data partitions, and 40 training seeds; the only intended difference was the frame-selection strategy.

### 3.7. Statistical Analysis

For the downstream diagnostic evaluation, each model configuration was trained independently 40 times using different random seeds while the data partitions and held-out 46-video test set were fixed. The same seed set was used across conditions. Statistical inference was performed at the patient level rather than at the run or video level. For each of the 34 held-out patients and each condition, correctness was first averaged across all contributed ears/videos and all 40 runs, producing one patient-level accuracy value per condition. The reported mean accuracy is the equally weighted mean of these 34 patient-level values, and the between-patient standard deviation is their sample standard deviation. Condition-specific 95% confidence intervals were estimated using a nonparametric bootstrap with 200,000 resamples of the 34 patients. The two primary matched comparisons were LUCID Top-1 versus the single expert-selected frame and LUCID Top-12 versus Uniform-12. For these two paired contrasts, two-sided exact patient-level sign-flip tests were used, and the corresponding 95% confidence intervals for the paired differences were obtained by inversion of the same exact sign-flip tests so that interval estimates and hypothesis tests were inferentially aligned. Secondary Top-12 comparisons with Top-1, Top-4, Top-16, and all-frame conditions were also evaluated using exact two-sided patient-level sign-flip tests, with Holm adjustment across these four secondary comparisons. The Top-12-versus-Human contrast was retained only as an overall workflow comparison because the conditions differ in both frame count and aggregation. In addition to the patient-level inferential analysis, run-to-run accuracy distributions across the 40 training realizations were examined descriptively as a secondary assessment of training stability. Exploratory two-sided exact two-sample Kolmogorov-Smirnov tests with tie-aware *p*-value calculation were used only to characterize differences among the run-level accuracy distributions. These run-level analyses are presented and discussed in Section 4.3 and were not used as primary clinical inferential evidence.

## 4. Results

### 4.1. Eardrum Visibility Classification Results

Table 2 presents the confusion matrix for the three-class classification task (“no eardrum,” “partial eardrum,” and “full eardrum”) on the test dataset.

The held-out test set contained 13,527 frames from 51 videos and 45 patients, and 13,489 frames were correctly classified (frame-level accuracy, 99.72%). All 38 errors occurred in two videos from two different patients: 24 of 267 frames were misclassified in one video and 14 of 318 in the other; the remaining 49 test videos had no frame-level errors. As a dependence-aware sensitivity analysis, accuracy was first calculated within each of the 51 held-out videos and the 51 video-level accuracies were then bootstrapped with 200,000 video-level resamples. The mean video-level accuracy was 99.74% (95% bootstrap CI, 99.30–100.00%). The very high accuracy should be interpreted in the context of the annotation protocol: visually ambiguous partial-versus-full frames were excluded before model development rather than assigned a forced label.

### 4.2. Eardrum Segmentation Results

As described in Section 3.3, the Eardrum Coverage Score requires localization of the tympanic membrane. Segmentation performance was evaluated on the held-out manual reference subset of 764 frames from 36 test videos and 31 patients described in Section 3.6. Two otolaryngologists (ACM and CE) independently delineated masks using X-AnyLabeling and subsequently reconciled them by discussion and joint editing to obtain a consensus reference for each frame. Table 3 compares BC-AdvCAM with Grad-CAM, Adv-CAM, and SAM using different numbers of positive point prompts (SAM_p_0 to SAM_p_3). The positive point prompts were randomly selected within the expert reference region. Evaluation metrics included IoU, DSC, pixel accuracy, sensitivity, specificity, AUROC, Hausdorff distance (HD), and mean absolute deviation (MAD).

To compare segmentation methods while accounting for repeated frames within patients, generalized estimating equations (GEE) with a sandwich variance estimator, exchangeable working correlation structure, and identity link were fit to 764 frame-level observations clustered within 31 patients, as summarized in Table 4.

BC-AdvCAM achieved higher IoU, DSC, accuracy, specificity, and lower HD than Adv-CAM, whereas Adv-CAM achieved higher sensitivity, higher AUROC, and lower MAD. Relative to Grad-CAM, BC-AdvCAM achieved higher IoU, DSC, accuracy, specificity, and AUROC, while the sensitivity difference was not statistically significant in the GEE analysis. Thus, the results indicate a metric-dependent trade-off rather than uniform superiority of BC-AdvCAM across all segmentation measures.

### 4.3. Most Informative Frame Selection Results

As described in Section 3.1, two ENT experts jointly reviewed each of the 303 evaluation videos and selected one reference MIF by consensus. We then applied LUCID to the same videos to obtain automatically selected MIFs. We evaluated the expert-selected and algorithm-selected MIFs using two complementary approaches.

**Figure 4:**
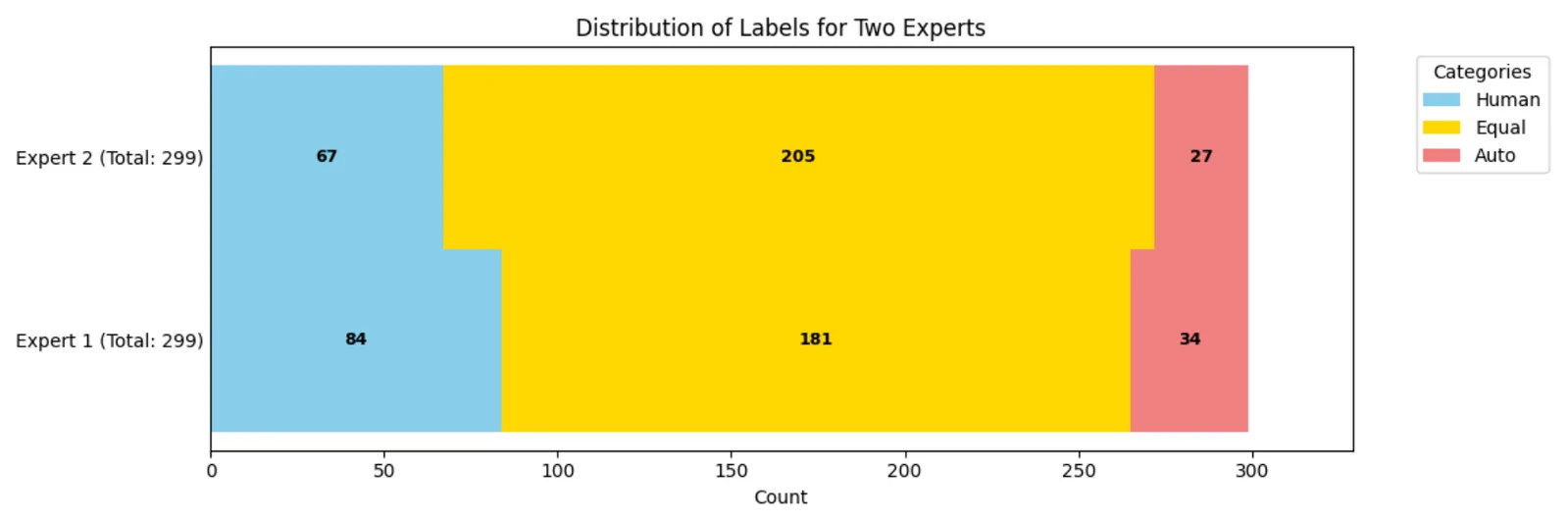
Human Assessment of MIF Selection. This stacked bar chart illustrates the independent human evaluation of automatically selected (Auto) versus expert-selected (Human) Most Informative Frames (MIFs) from 299 otoscopy videos. Blue indicates reviewer preference for the expert-selected MIF, red for the algorithm-selected MIF, and yellow for cases where both were considered equivalent or indistinguishable.

**Figure 5:**
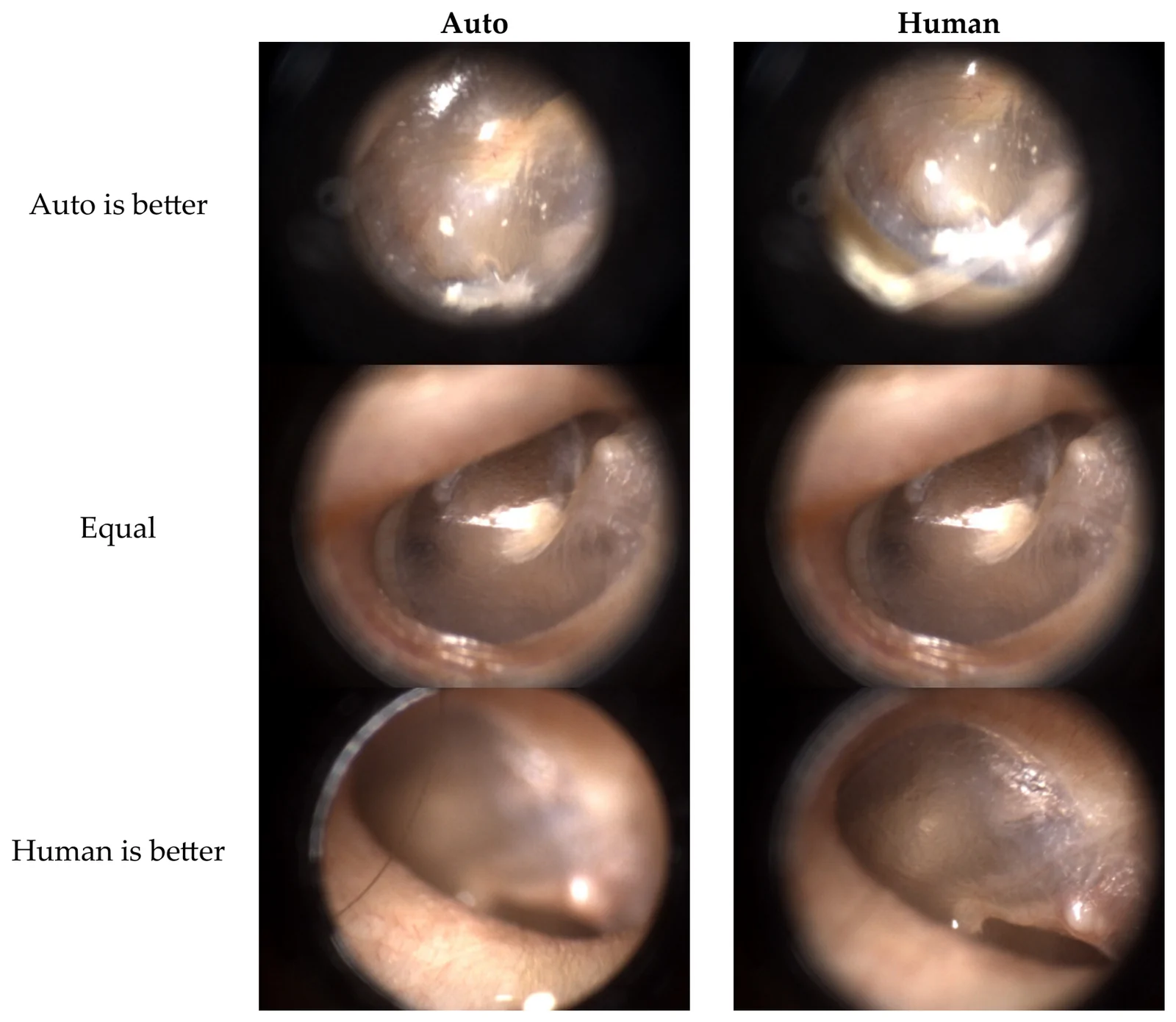
Representative examples comparing automatically selected and expert-selected Most Informative Frames (MIFs). Each row shows a paired comparison from the same otoscopy video. Examples illustrate cases in which the algorithm-selected frame was preferred, the two frames were judged equivalent in informativeness, and the expert-selected frame was preferred by the human evaluators.

An emergency physician and a trained medical student, both with otoscopy training, independently evaluated 299 pairs of MIFs, each comprising one expert-selected and one algorithm-selected frame (four videos were excluded because of transmission errors). The evaluators were fully blinded to frame-selection source, and the left-right position of the expert-selected and algorithm-selected frames was randomized independently for each pair. Of the 299 evaluated pairs, 293 had complete valid ratings from both evaluators and were included in the inter-rater agreement analysis. Among these 293 pairs, the algorithm-selected frame was rated equal to or better than the expert-selected frame in 77.5% of ratings by Demir and 72.0% by Gabriella (74.7% on average). The results are summarized in Figure 4 and Table 5 and Table 6. Representative Auto-better, Equal, and Human-better paired examples are shown in Figure 5.

**Table 5:** Confusion matrix of blinded evaluator preferences for the 293 video pairs with complete ratings. Rows = Demir; columns = Gabriella.

Demir\Gabriella	Human	Auto	Equal	Total
Human	44	6	16	66
Auto	4	11	11	26
Equal	34	17	150	201
Total	82	34	177	293

**Table 6:** Human-versus-Auto preference among non-Equal ratings.

Evaluator/Subset	Human	Auto	Not Sure	Human %	Two-Sided Exact *p*-Value
Demir	66	26	92	71.7%	3.67 × 10^−5^
Gabriella	82	34	116	70.7%	9.69 × 10^−6^
Both agreed non-Equal	44	11	55	80.0%	8.70 × 10^−6^

Second, we trained four common image classification models (ResNet34, ConvNeXt-Tiny, Swin Transformer-Tiny, and ViT-B/16) for four-class otoscopy diagnosis (Normal, Effusion, Perforation, and Tympanosclerosis). The 303-video evaluation cohort was partitioned at the patient level into 227 training videos from 142 patients, 30 validation videos from 19 patients, and 46 test videos from 34 patients. All videos from a given patient were kept within a single split to prevent information leakage between ears from the same individual. Each architecture was trained separately using either the expert-selected MIFs or the algorithm-selected MIFs. Bayesian optimization was performed for 40 iterations using only the validation set to tune hyperparameters.

**Figure 6:**
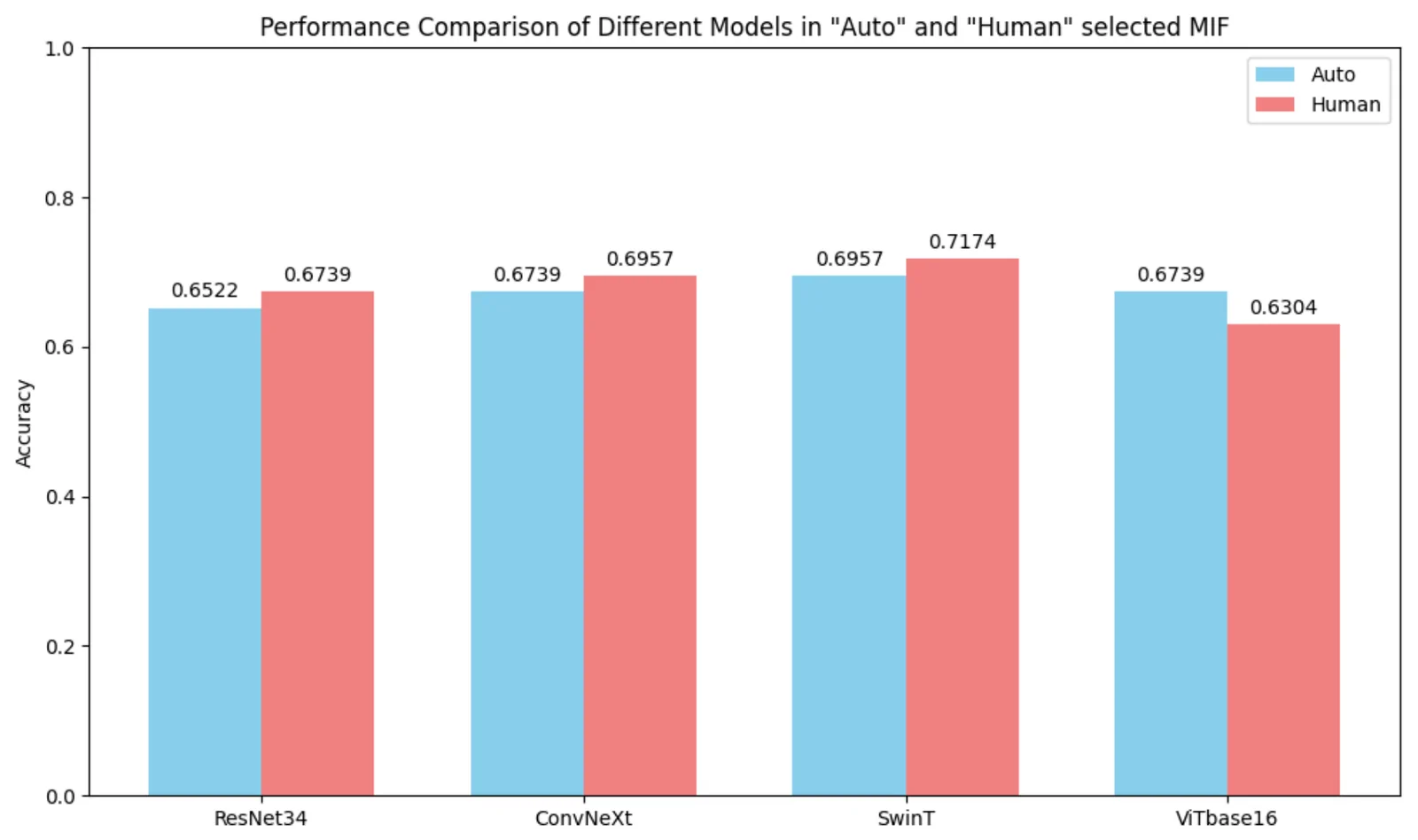
Comparative Performance of Automated Diagnostic Models Trained on Different MIF Sources. Red bars indicate the expert-selected (Human) condition, and blue bars indicate the automatically selected (Auto) condition.

Figure 6 illustrates the observed performance of automated diagnostic models trained on expert-selected versus algorithm-selected MIFs. The models trained on automatically selected MIFs showed similar observed accuracy to those trained on expert-selected MIFs. For ResNet34, ConvNeXt-Tiny, and Swin Transformer-Tiny, training with algorithm-selected MIFs resulted in an approximately 2% lower accuracy than training with expert-selected MIFs, whereas ViT-B/16 showed a higher observed accuracy with algorithm-selected MIFs. Multi-class accuracy reflects the proportion of correctly classified test images across the four diagnostic categories. The fact that Auto was lower than Human for three of the four architectures is consistent with the matched single-frame result reported below and is not interpreted as evidence that LUCID Top-1 surpasses expert single-frame selection.

The expert reference task produced one consensus representative frame per video; obtaining a complete human ranking of 12 or 16 frames from every video would require a substantially different and much more burdensome annotation protocol. LUCID instead ranks the entire video automatically, which makes construction of ranked multi-frame bags practical. To test whether the observed multi-frame benefit reflects LUCID ranking rather than simply using more frames, we added Uniform-12 as a matched 12-frame control using uniform temporal sampling and the identical ResNet-50 + ABMIL pipeline.

For the ranked-frame analysis, Top-12 remained the k value selected exclusively on the classification validation set (validation accuracies: Human 0.80, Top-1 0.68, Top-4 0.76, Top-12 0.83, Top-16 0.71, and all frames 0.57) and was locked before held-out testing. Primary test-set inference was performed at the patient level by first averaging correctness within each of the 34 patients across all contributed ears and 40 training runs. Patient-level mean accuracies were 0.722 for Human, 0.688 for Top-1, 0.750 for Top-4, 0.781 for Top-12, 0.594 for Uniform-12, 0.704 for Top-16, and 0.642 for all frames. Between-patient standard deviations and patient-bootstrap confidence intervals are reported in Table 7.

**Figure 7:**
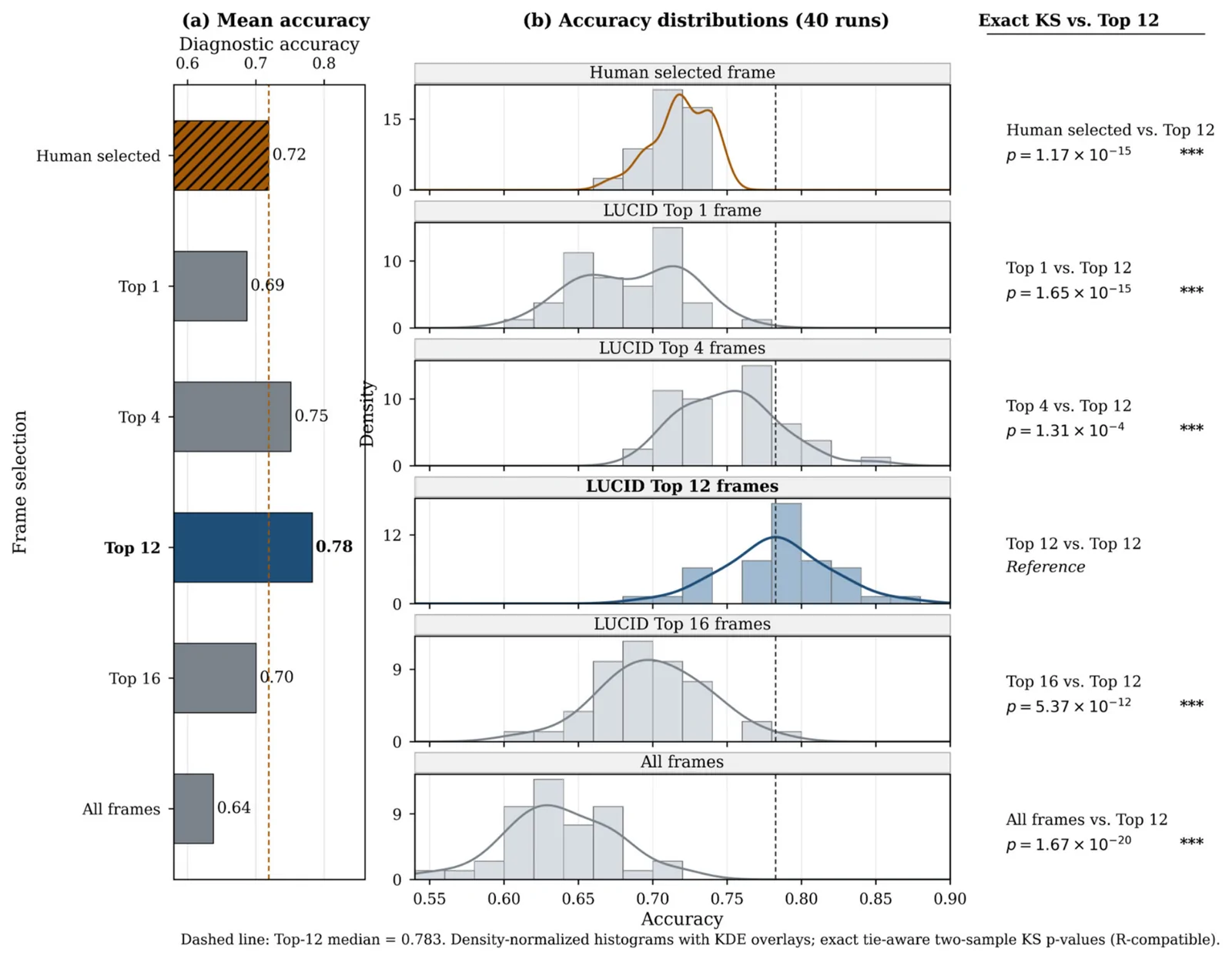
Descriptive run-to-run diagnostic performance across 40 independently seeded training realizations for the original frame-selection conditions. Panel (**a**) shows video-weighted run-level mean accuracy and panel (**b**) shows density-normalized run-level accuracy distributions with kernel density estimates. The dashed line indicates the Top-12 median run-level accuracy. The KS *p*-values displayed in the right panel are exploratory run-level distributional comparisons and are not used for patient-level inference. Primary inference is based on equally weighted patient-level estimates and matched patient-level comparisons in Table 7.

Figure 7 provides a descriptive visualization of run-to-run training variability for the original Top-k conditions. Because the same 34 held-out patients are reused in all 40 runs, the run-level distributions and exploratory KS results are not used as primary inferential evidence. The patient-level estimates, between-patient standard deviations, and matched comparisons, including the Uniform-12 control, are reported in Table 7.

At matched single-frame count, LUCID Top-1 had a patient-level mean accuracy of 68.79% versus 72.17% for the expert-selected frame, a difference of −3.38 percentage points (95% exact sign-flip-inversion CI, −6.79 to 0.00; two-sided exact patient sign-flip *p* = 0.0540). Thus, the present data do not support a claim that LUCID Top-1 outperforms expert single-frame selection. In contrast, the matched 12-frame comparison directly isolates frame selection while holding bag size and ABMIL constant: LUCID Top-12 achieved 78.13% versus 59.41% for Uniform-12, a +18.71-percentage-point difference (95% exact sign-flip-inversion CI, +15.79 to +21.63; *p* = 2.33 × 10^−10^). The overall Top-12-versus-Human difference was +5.96 percentage points (95% CI, +2.90 to +8.93), but this contrast is not interpreted as a pure ranking effect because the two conditions differ in frame count and aggregation. For the four secondary Top-12 comparisons, Holm-adjusted *p*-values were 2.46 × 10^−5^ versus Top-1, 0.04476 versus Top-4, 4.89 × 10^−5^ versus Top-16, and 2.42 × 10^−7^ versus all frames.

### 4.4. Human Preference Analysis

To evaluate human preference and inter-rater agreement without assuming an equal 1:1:1 distribution across Human, Auto, and Equal responses, we analyzed the 293 video pairs with complete ratings from both evaluators. Exact agreement occurred in 205 of 293 pairs (69.97%). Cohen’s kappa was 0.414, with a 95% nonparametric bootstrap percentile confidence interval of 0.318–0.506 based on 20,000 resamples, indicating moderate inter-rater agreement. The cross-classification of the two evaluators’ ratings is shown in Table 5.

Preference direction was evaluated after excluding Equal ratings. For Demir, 66 of 92 non-Equal ratings (71.7%) favored the expert-selected Human frame and 26 favored Auto; a two-sided exact binomial test against a 50:50 preference yielded *p* = 3.67 × 10^−5^. For Gabriella, 82 of 116 non-Equal ratings (70.7%) favored Human and 34 favored Auto (two-sided exact *p* = 9.69 × 10^−6^). Among the 55 video pairs for which both evaluators agreed on the same non-Equal category, 44 (80.0%) favored Human and 11 favored Auto (two-sided exact *p* = 8.70 × 10^−6^). These results are summarized in Table 6.

Overall, the evaluators showed moderate agreement. When a directional preference was expressed rather than an Equal rating, both evaluators more often selected the expert-selected Human frame; among pairs for which both evaluators agreed on the same non-Equal category, 80.0% favored Human. Representative paired examples across the three preference outcomes are shown in Figure 5. This subjective single-frame preference is consistent with the matched Top-1-versus-Human diagnostic result and should be interpreted separately from LUCID’s ability to construct ranked multi-frame evidence sets.

Overall exact agreement = 205/293 (69.97%); Cohen’s kappa = 0.414; 95% bootstrap percentile CI = 0.318–0.506 (20,000 resamples).

## 5. Discussion

The primary aim of this study was to develop and evaluate a systematic informative-frame selection approach for otoscopy video sequences. This addresses a practical limitation of many otoscopy AI workflows that rely on manually selected still frames. The present results support the feasibility of the proposed ranking strategy and motivate further validation, while the single-institution, single-device design limits conclusions about broader clinical utility.

The revised analyses separate two distinct questions that were previously conflated. First, for the matched single-frame task, the expert-selected frame had the higher observed patient-level mean accuracy: LUCID Top-1 achieved 68.79% patient-level mean accuracy versus 72.17% for the expert-selected frame (difference, −3.38 percentage points; 95% exact sign-flip-inversion CI, −6.79 to 0.00; two-sided exact sign-flip p = 0.0540). This direction is also consistent with the human-preference analysis and with Figure 6, where Auto was lower than Human for three of four single-frame architectures. Second, LUCID is designed to rank the entire video rather than only return a single frame. A human-generated ranking of 12 or 16 frames per examination was not available and would require a substantially more burdensome annotation task. We therefore added a matched Uniform-12 control to isolate ranking from bag size and ABMIL. With the same 12-frame bag size, architecture, splits, and training seeds, LUCID Top-12 achieved 78.13% versus 59.41% for Uniform-12, a +18.71-percentage-point difference (95% exact sign-flip-inversion CI, +15.79 to +21.63; *p* = 2.33 × 10^−10^). This matched-k result supports the contribution of LUCID ranking beyond multi-frame aggregation alone. The higher overall Top-12 accuracy relative to the single Human condition is reported descriptively but is not used to attribute an effect solely to ranking because frame count and aggregation differ between those conditions.

To place LUCID in context, Table 8 compares representative otoscopy and related endoscopic frame-selection approaches. The comparison emphasizes that automated frame filtering is not itself new: prior work has used anatomical segmentation, valid-frame detection, composite-image generation, direct video modeling, supervised informative-frame classification, and redundancy removal [13,14,15,16,17,18,19,20,21,22,23].

The comparison also clarifies what is and is not new in LUCID. Automatic frame selection, eardrum localization, blur assessment, multi-frame video representation, and deep video models have all been reported previously [13,15,16,17,18,19,20,21,22,23]. LUCID’s contribution lies instead in combining three otoscopy-specific, separately interpretable criteria—tympanic-membrane visibility, tympanic-membrane coverage, and blur/focus—into a unified within-video ranking, together with background-suppressed weakly supervised localization and evaluation at both the human-selected-MIF and downstream Top-k diagnostic levels. The individual building blocks (ResNet, class-activation mapping, Hough-circle detection, gradient-based sharpness analysis, and rank fusion) are established techniques; the contribution is their task-specific integration and validation rather than novelty of each component.

OtoSTVT [19] is the closest contemporary comparator because it directly learns diagnostic frame relevance from temporal context. The two methods represent different design choices rather than a simple superiority relationship. OtoSTVT uses an end-to-end spatio-temporal Transformer trained with dense ordinal relevance labels, whereas LUCID decomposes informativeness into explicit anatomical and image-quality factors and can be trained without dense ENT relevance annotation for every frame. This makes LUCID more transparent at the component-score level, but it does not explicitly model temporal dependencies and its fusion weights are selected on validation data rather than learned end-to-end. Moreover, unlike externally validated laryngoscopy quality-selection systems [21,22], LUCID has not yet been validated across institutions or otoscope devices. These limitations define the appropriate scope of the present claims and motivate external and temporal-model validation in future work.

**Figure 8:**
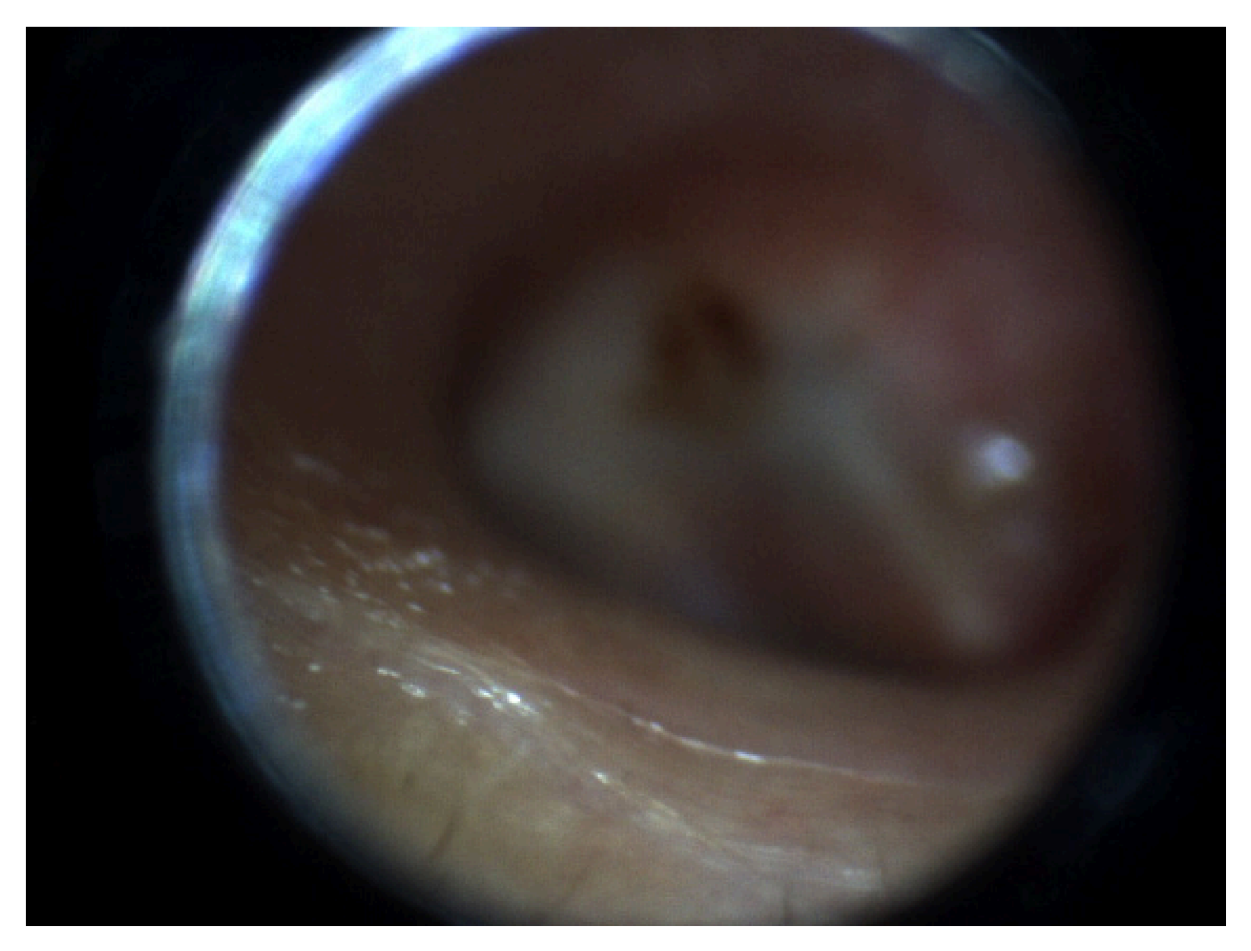
Example of Algorithm Failure in MIF Selection.

Several limitations should be considered. The three medical-student annotators labeled separate assigned subsets after a one-week training program rather than redundantly labeling the same frames, and the two ENT experts selected reference MIFs jointly by consensus; therefore, inter-rater reliability could not be estimated for those annotation tasks. The near-perfect Eardrum View classification accuracy should also be interpreted in the context of its intended role as a coarse quality-control task: visually ambiguous partial-versus-full frames were excluded during annotation, so the reported test performance does not establish performance on borderline visibility cases. For segmentation, the 764 manually segmented frames were restricted to 36 videos from 31 patients in the held-out development test split and were never used for training or validation. The two otolaryngologists initially delineated the segmentation masks independently and then reconciled differences to a single consensus mask; a formal inter-rater segmentation reliability analysis was not reported. Our dataset was collected at a single institution using one otoscope model, so external validation across institutions, devices, and populations remains necessary. BC-AdvCAM performance was metric-dependent: it improved overlap, accuracy, specificity, and HD relative to Adv-CAM, whereas Adv-CAM retained advantages in sensitivity, AUROC, and MAD. A representative LUCID frame-selection failure case is shown in Figure 8.

Looking ahead, we envision this research serving as a foundational auxiliary tool to enhance the capabilities of automated otoscopy diagnosis. Simultaneously, it can function as an assistance tool for annotating otoscopy MIFs, enabling experts to label and build larger, more comprehensive datasets rapidly. Such datasets, in turn, can facilitate the training of even more precise and robust MIF selection models in the future.

Clinical Implications: By automatically highlighting diagnostically useful frames, LUCID may help reduce the amount of video that requires manual review and may support annotation and quality-assurance workflows. These potential clinical benefits were not directly measured in the present study. Prospective workflow studies and external validation across institutions and device types are required before conclusions can be drawn regarding review-time reduction, real-time use, or clinical deployment.

## 6. Conclusions

This study developed and evaluated a systematic framework for informative-frame ranking from otoscopy videos. The revised matched analyses distinguish single-frame selection from multi-frame evidence construction and therefore provide a more specific interpretation of LUCID’s contribution.

For single-frame selection, the expert reference had the higher observed patient-level mean accuracy: LUCID Top-1 achieved 68.79% versus 72.17% for Human, and directional human preferences also favored the expert frame. In contrast, when bag size and ABMIL were matched at 12 frames, LUCID Top-12 achieved 78.13% versus 59.41% for Uniform-12 (difference, +18.71 percentage points; 95% exact sign-flip-inversion CI, +15.79 to +21.63; *p* = 2.33 × 10^−10^). Thus, the principal evidence for LUCID is not that its single Top-1 frame surpasses an expert-selected frame, but that automatic ranking of the full video provides a substantially more informative 12-frame evidence set than uniform sampling under an otherwise identical multi-frame pipeline. External validation and prospective workflow evaluation remain necessary before claims of clinical utility or deployment can be made.

Additional limitations include the use of weakly supervised localization for coverage estimation, potential failure of the blur/focus module under specular reflection, wax occlusion, or off-center focus, and the absence of external device or institutional validation. Future work should evaluate borderline visibility cases rather than excluding them, include independent redundant annotations where feasible, and test the ranking framework in larger multi-center cohorts.

For future work, we plan to integrate temporal information across video frames to enhance the robustness of frame selection. Additionally, we aim to incorporate clinical metadata to guide the learning process further. Expanding expert annotation efforts will also enable us to train fully supervised segmentation models and improve blur detection using more advanced self-supervised or foundation models. Ultimately, we envision incorporating this framework into real-time otoscope workflows to provide on-the-fly frame selection and diagnostic support.

## Figures and Tables

**Table 1:** Patient-level dataset partitions and diagnostic category distribution. Part. = partition; Vid. = videos; Pt. = patients; Ped. = pediatric; Norm. = Normal; Eff. = Effusion; TS = Tympanosclerosis; PF = Perforation. Patient, Adult, and Pediatric counts are patient-level counts, whereas the four diagnostic-category counts are video/ear-level counts.

Part.	Vid.	Pt.	Adult	Ped.	Norm.	Eff.	TS	PF
MIF Train	296	207	169	38	134	106	28	28
MIF Val	63	44	36	8	29	23	6	5
MIF Test	51	45	36	9	23	18	5	5
MIF Total	410	296	241	55	186	147	39	38
Cls Train	227	142	116	26	103	82	22	20
Cls Val	30	19	15	4	14	11	3	2
Cls Test	46	34	28	6	20	16	4	6
Cls Total	303	195	159	36	137	109	29	28
Overall	713	491	400	91	323	256	68	66

**Table 2:** Confusion matrix for Eardrum View classification on the held-out test set. Cells show count (row percentage); totals are counts.

True Label	Pred. no Eardrum	Pred. Partial Eardrum	Pred. Full Eardrum	Total
No eardrum	1681 (99.41%)	4 (0.24%)	6 (0.35%)	1691
Partial eardrum	15 (0.45%)	3297 (99.40%)	5 (0.15%)	3317
Full eardrum	8 (0.09%)	0 (0.00%)	8511 (99.91%)	8519
Total	1704	3301	8522	13,527

**Table 3:** Comparison of segmentation performance for different methods, including Grad-CAM, Adv-CAM, our proposed BC-AdvCAM, and SAM with varying positive point prompts (SAM_p_0 to SAM_p_3). ↑ indicates that larger values are better, and ↓ indicates that smaller values are better.

Method	IoU ↑	DSC ↑	Acc ↑	Sen ↑	Spe ↑	AUROC ↑	HD ↓	MAD ↓
Grad-CAM	0.5436	0.6753	0.8805	0.7834	0.8969	0.8681	38.4291	1.8078
Adv-CAM	0.6176	0.7297	0.8858	0.8795	0.8760	0.9128	35.2936	0.3229
BC-AdvCAM	0.6717	0.7924	0.9091	0.8546	0.9282	0.8908	32.2179	1.1937
SAM_p_0	0.0911	0.1364	0.4258	0.3101	0.4553	0.4288	124.2249	38.1030
SAM_p_1	0.3704	0.5193	0.5799	0.9987	0.4591	0.7172	91.2550	0.0029
SAM_p_2	0.4088	0.5603	0.6521	0.9989	0.5511	0.7477	83.8071	0.0058
SAM_p_3	0.4338	0.5859	0.6911	0.9996	0.5999	0.7655	78.7163	0.0008

**Table 4:** Generalized Estimating Equation (GEE) analysis of segmentation metrics, including pixel-level AUROC, using 764 frame-level observations clustered within 31 patients, with an exchangeable correlation structure and identity link.

Metric	Delta = BC_AdvCAM—X	Estimate	Estimate SE	*p*-Value
IoU	Adv-CAM	0.0466	0.0164	0.0045
IoU	Grad-CAM	0.0882	0.0158	2.47 × 10^−8^
DSC	Adv-CAM	0.0384	0.012	0.00139
DSC	Grad-CAM	0.0671	0.0122	3.93 × 10^−8^
Accuracy	Adv-CAM	0.0341	0.0065	1.59 × 10^−7^
Accuracy	Grad-CAM	0.0352	0.0056	3.16 × 10^−10^
Sensitivity	Adv-CAM	−0.1227	0.0109	<2 × 10^−16^
Sensitivity	Grad-CAM	0.0102	0.0178	0.56
Specificity	Adv-CAM	0.0797	0.0064	<2 × 10^−16^
Specificity	Grad-CAM	0.0371	0.0059	4.10 × 10^−10^
AUROC	Adv-CAM	−0.0215	0.0065	9.80 × 10^−4^
AUROC	Grad-CAM	0.0237	0.0085	0.00552
HD	Adv-CAM	−7.4465	1.9005	8.92 × 10^−5^
HD	Grad-CAM	−6.4042	1.2581	3.58 × 10^−7^
MAD	Adv-CAM	1.0975	0.1699	1.06 × 10^−10^
MAD	Grad-CAM	−0.5793	0.2849	0.0420

**Table 7:** Patient-level diagnostic accuracy on the held-out test cohort. For each condition, correctness was first averaged within each of the 34 patients across all contributed ears and 40 training runs; patients were then treated as equal-weighted analysis units. SD = sample standard deviation across the 34 patient-level accuracies. Condition-specific confidence intervals are nonparametric patient-bootstrap percentile intervals based on 200,000 resamples. Confidence intervals for the two primary paired differences are obtained by inversion of the corresponding exact two-sided patient-level sign-flip tests.

Condition	Patient-Level Mean Accuracy	Between-Patient SD	Patient-Bootstrap 95% CI	Primary Matched Comparison *
Human	0.722	0.068	0.700–0.744	Reference for Top-1
Top 1 frame	0.688	0.066	0.666–0.710	vs Human: Delta = −0.0338 95% exact sign-flip-inversion CI: −0.0679 to 0.0000 *p* = 0.0540
Top 4 frames	0.750	0.067	0.728–0.772	-
Uniform-12	0.594	0.058	0.575–0.614	Reference for Top-12
Top 12 frames	0.781	0.062	0.761–0.801	vs Uniform-12: Delta = +0.1871 95% exact sign-flip-inversion CI: +0.1579 to +0.2163 *p* = 2.33 × 10^−10^
Top 16 frames	0.704	0.051	0.688–0.722	-
All frames	0.642	0.083	0.614–0.669	-

* Note: The two primary matched comparisons are Top-1 versus Human (one frame versus one frame) and Top-12 versus Uniform-12 (12 frames versus 12 frames with the identical ABMIL pipeline). Reported *p*-values and paired-difference 95% confidence intervals for these contrasts are derived from the same exact two-sided patient-level sign-flip procedure. Secondary Top-12 comparisons with Top-1, Top-4, Top-16, and all frames were Holm-adjusted as a four-comparison family.

**Table 8:** Representative prior work related to informative-frame selection, video representation, and quality filtering in otoscopy and medical endoscopy.

Study/Year	Application/Input	Frame Selection/Representation	Supervision/Expert Benchmark	Downstream use/External Validation	Main Advantage/Limitation
Binol et al. [15], 2020	Otoscopy video	TM segmentation; >=20% TM-area threshold; stitching	Pixel-level TM masks; 3 ENT physicians graded composites	Selected frames -> composite; no external validation	Interpretable anatomical filtering; no ranked MIF and depends on threshold/stitching.
Wang et al. [18], 2022	Pediatric otoscopy video	Eardrum-patch detection + frame anomaly-score aggregation	Supervised detector + self-supervised representation; clinician screening comparison	Normal/abnormal video screening; no external validation	Uses real videos and few abnormal labels; valid-frame filtering is not explicit MIF ranking.
Binol et al. [16], 2022	Otoscopy video	Multiple composite images as video representation	Disease labels; human keyframe baseline	Multi-class diagnosis; no external validation	Improves video representation; does not output frame-informativeness ranks.
Yao et al. [20], 2022	Flexible laryngoscopy frames	ResNet-18 informative/uninformative classifier	Two-reviewer frame labels; agreement assessed	Frame filtering; no external validation	Explicit visibility/lighting/focus criteria; different anatomy and binary label.
Baldini et al. [21], 2024	Laryngeal endoscopy frames	Real-time DL informative/uninformative classification	Multi-reviewer frame labels	Real-time selection; external validation	Externally validated and real-time; relevance/quality task rather than otoscopy MIF ranking.
Lu et al. [17], 2025	Otoscopy video clips	VideoMAE sampled clips; no explicit MIF ranking	Disease labels	Direct 7-class video diagnosis; no external validation	Avoids manual still-frame selection; no interpretable frame ranking.
Camalan et al. [14], 2025	Otoscopy: composite/still/video	Clinical comparison of visualization formats	Expert diagnosis; 9-ENT reader study	Clinical accuracy/time comparison	Direct clinical comparison; not a new MIF-ranking algorithm.
Camalan et al. [19], 2026	Otoscopy video	Diagnostic-driven spatio-temporal Transformer (OtoSTVT)	Three-point ordinal relevance labels from trained reviewers	Ranks/selects frames; no external validation	Learns temporal relevance end-to-end; requires dense relevance labels; reported F1 = 0.62.
Kim et al. [22], 2026	Laryngoscopy images	Three-tier image-quality classification	Expert quality labels	High-quality image selection; external validation	Externally validated quality selection; quality is not equivalent to diagnostic MIF ranking.
Li et al. [23], 2026	Capsule endoscopy video	Triplet-network redundant-frame removal	Similarity/redundancy learning; clinician keyframe evaluation	Video de-redundancy; no external validation	Large-scale efficient de-redundancy; similarity objective differs from diagnostic relevance.
LUCID, this study	Otoscopy video	Rank fusion of TM visibility, coverage, and blur/focus	Student view labels + weak supervision; ENT consensus MIF + blinded preference study	Top-k ABMIL + matched Uniform-12 control; 4-class diagnosis; no external validation	Interpretable decomposed ranking with matched-k downstream validation; no temporal modeling and single-site/device evaluation

## Data Availability

The datasets generated and/or analyzed during the current study are not publicly available due to institutional and patient privacy regulations under the Wake Forest University Health Sciences Institutional Review Board (IRB0004833). The data contain identifiable medical information that cannot be fully anonymized. However, de-identified data may be available from the corresponding author on reasonable request and with appropriate institutional approvals. The source code is available at https://github.com/CAIR-LAB-WFUSM/LUCID-Informative-Frame-Selection-in-Otoscopy-.git (accessed on 7 September 2026).
